# Applications and future prospects of laser technologies in the treatment of non-muscle invasive bladder cancer

**DOI:** 10.14440/bladder.2025.0004

**Published:** 2025-06-05

**Authors:** Yiting Liu, Lilong Liu, Zhipeng Yao, Yaxin Hou, Zhenghao Liu, Yang Li, Yuhong Ding, Pengjie Shi, Zheng Liu, Ke Chen

**Affiliations:** 1 Department of Urology, Tongji Hospital of Tongji Medical College, Huazhong University of Science and Technology, Wuhan 430030, China; 2 Institute of Urology, Tongji Hospital of Tongji Medical College, Huazhong University of Science and Technology, Wuhan, Hubei 430030, China

**Keywords:** Bladder cancer, Laser, Prognosis

## Abstract

**Background:**

Bladder cancer (BCa) represents a common malignancy of the urinary system across the globe and poses significant treatment challenges.

**Objective:**

This review comprehensively examined the application of laser technology in the treatment of BCa, including thulium (Tm) lasers (e.g., Tm-doped yttrium aluminium garnet [YAG]), GreenLight lasers (e.g., potassium titanyl phosphate-doped YAG and lead aluminum borate-doped YAG), holmium lasers (e.g., Ho-doped YAG), Tm fiber laser (TFL), and diode lasers. Studies have demonstrated that laser technology effectively improves tumor resection quality, reduces intraoperative bleeding, shortens recovery time, and lowers recurrence rates. Among these, Ho-doped YAG and Tm-doped YAG lasers have been shown to reduce tissue damage and enhance resection precision, while the TFL has attracted attention for its efficient tissue cutting and minimal thermal damage. GreenLight lasers offer advantages in preserving surrounding healthy tissues and demonstrate potential for use in outpatient settings. Diode lasers, known for their high-energy efficiency, contribute to improved overall treatment efficacy. This article further explored the benefits, drawbacks, and prospective uses of each laser technology in BCa treatment.

**Conclusion:**

This article thoroughly reviewed the applications of various laser technologies in BCa treatment, highlight their benefits and limitations, and assess their potential roles in future clinical practice.

## 1. Introduction

Bladder cancer (BCa) is one of the most common malignancies of the urinary system, with roughly 573,000 new cases and 213,000 deaths reported annually.^1^ Non-muscle invasive BCa (NMIBC) accounts for approximately 75% of BCa cases and is characterized by a high recurrence rate – ranging from 31% to 78% within 5 years – and up to 45% of cases may progress to muscle-invasive BCa (MIBC).^2^-^4^

Laser technology has emerged as a promising approach for NMIBC treatment, attaining precise tumor resection, reduced intraoperative bleeding, and lower recurrence rates. The continued development of urological laser techniques, including hybrid laser systems and outpatient-based laser treatments, further improves surgical outcomes and promotes patient recovery.^1^,^5^-^7^

Using PubMed (https://pubmed.ncbi.nlm.nih.gov/), we identified and analyzed published studies focusing on the clinical application of the most commonly used lasers for the treatment of NMIBC, including holmium (Ho) lasers (e.g., Ho-doped yttrium aluminium garnet [YAG]), thulium (Tm) lasers (e.g., Tm: YAG), GreenLight lasers (potassium titanyl phosphate [KTP]-doped YAG] and lead aluminum borate [LBO]-doped YAG]), Tm fiber laser (TFL), and diode lasers. Studies involving other laser modalities, as well as those focusing on MIBC or metastatic disease, were excluded to ensure a targeted and relevant analysis. This selection criteria aimed to provide a comprehensive evaluation of laser-based interventions specifically for NMIBC, in line with current clinical practices and surgical advancements.

**Figure 1:**
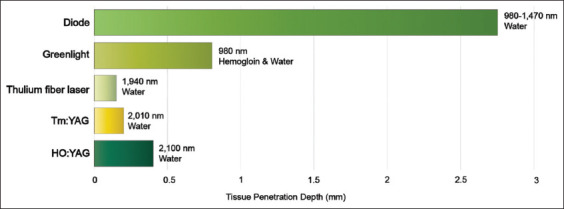
Comparison of laser-based technologies in bladder cancer treatment Abbreviations: Ho:YAG: Holmium-doped yttrium aluminium garnet; KTP: Potassium titanyl phosphate; LBO: Lead aluminum borate; Tm:YAG: Thulium-doped yttrium aluminium garnet.

Therefore, this review thoroughly evaluated the applications of various laser technologies in BCa treatment, highlight their benefits and limitations, and assess their potential future roles in clinical settings (Table 1 and Figure 1). By analyzing current literature, this article intended to support the enhancement and innovation of BCa therapies.

**Table 1:** A comparative overview of laser technologies in bladder cancer treatment

Laser type	Wavelength (μm)	Mode of action	Absorption coefficient in water (cm^−1^)	Theoretical tissue penetration depth (mm)	Advantages	Disadvantages
Ho:YAG	2.10	Pulsed	26	0.4	Provides excellent hemostasis and precise cutting, and is widely used in external beam radiation therapy.	May cause mechanical damage to tissues with pulsed mode.
Tm:YAG	2.01	Continuous	52	0.2	High tissue vaporization rate and low bleeding risk.	Risk of carbonization in continuous mode with limited tissue penetration depth.
Thulium fiber laser	1.94	Pulsed	114	0.15	Minimal thermal damage with highly precise cutting and low carbonization.	-
GreenLight (KTP:YAG or LBO:YAG)	0.53	Continuous	-	0.8	Provides excellent hemostasis and is widely used for soft-tissue cutting with minimal damage to surrounding tissues.	Wider incision margin, limited tissue penetration depth, and complete resection of tumor tissue, but with high equipment costs.
Diode (980 nm or 1,470 nm)	0.98 – 1.47	Continuous	0.43 – 7.2	0.5 – 5.0	Deep tissue penetration, low cost, and high-energy efficiency.	-

Abbreviations: Ho:YAG: Holmium-doped yttrium aluminium garnet; KTP:YAG: Potassium titanyl phosphate-doped yttrium aluminium garnet; LOB:YAG: Lead aluminum borate-doped yttrium aluminium garnet; Tm:YAG, Thulium-doped yttrium aluminium garnet.

## 2. Overview of laser modalities in NMIBC treatment

This review explored the clinical applications and future prospects of various laser technologies in NMIBC treatment. It summarizes the clinical benefits, limitations, and potential developments in laser-based BCa therapies. The following sections introduce the Ho laser, Tm laser, TFL, GreenLight lasers, and diode lasers.

### 2.1. Ho laser

The Ho laser, such as the Ho:YAG laser, emits at a wavelength of 2,100 nm and exhibits high water absorption (26 cm^−1^), making it ideal for soft-tissue cutting and vaporization in urological applications. It achieves a tissue penetration range of 0.4 – 0.7 mm, allowing for precise removal of diseased tissues. Its high peak power (10 – 15 kW) and pulsed emission generate vapor bubbles that disrupt tissue effectively while ensuring excellent hemostasis.^8^,^9^

The Ho:YAG laser has become a key modality in BCa treatment thanks to its unique physical properties that enhance both safety and efficacy. Since its introduction for bladder tumor resection in 2001, multiple studies have validated its clinical benefits. Saito^10^ demonstrated that, in 35 patients involving 50 lesions, the Ho:YAG laser effectively precluded severe complications – such as uncontrollable bleeding or bladder perforation – while consistently yielding adequate pathological samples. In 2020, Maheshwari *et al*.^11^ reported that Ho:YAG laser bladder tumor resection in 67 NMIBC patients effectively addressed several limitations associated with traditional transurethral resection of bladder tumor (TURBT), including piecemeal resection and the occurrence of obturator nerve reflex (ONR). This technique also reduced the requirement for post-operative bladder irrigation and blood clot removal, increased the rate of muscle-positive specimens, and eliminated the need to convert to traditional TURBT. Similarly, Hashem *et al*.^12^ showed that Ho:YAG laser resection resulted in a significantly lower rate of post-operative residual tumors compared to TURBT (7% vs. 27.7%), while also accomplishing a higher rate of specimens containing bladder detrusor muscle (98% vs. 62%). Furthermore, patients in the Ho:YAG laser resection group experienced shorter catheterization and hospitalization durations, and post-operative chemotherapy was administered more smoothly. Although recurrence-free survival (RFS) did not differ significantly between groups, Ho:YAG laser resection exhibited conspicuous advantages in improving tumor resection quality and minimizing post-operative complications. Kramer *et al*.^13^ emphasized that the advantages of Ho:YAG laser extend beyond its effective hemostatic capabilities and its pulsed emission mode generates high-energy vapor bubbles that facilitate rapid tissue breakdown. In a multicenter European study comparing electrosurgical and laser-based bladder tumor resections, the electrosurgery group had a significantly higher conversion rate to traditional TURBT compared to its laser-treated counterpart (26.3% vs. 1.5%).^14^ In addition, a cost analysis conducted in Spain revealed that Ho:YAG laser resection was more cost-effective, with savings of €2,007.09 per procedure relative to transurethral cystectomy.^15^

Despite the widespread application of Ho:YAG laser due to its cutting precision, several studies have highlighted its potential drawbacks. Specifically, the pulsed output mode of Ho:YAG laser can cause excessive mechanical shock to tissues in certain cases, leading to tissue rupture or damage at the incision line. This underscores the need for surgeons to exercise particular caution during laser cutting, avoiding overly-concentrated or excessive focusing of the laser to prevent unnecessary tissue damage and ensure both safety and efficacy of the surgery.^13^ In addition, Li *et al*.^16^ noted that while the Ho:YAG laser offers clear advantages in cutting and hemostasis, it did not demonstrate significant superiority to traditional TURBT in controlling tumor recurrence for lesions smaller than 3 cm. This suggests that, while effective in certain areas, the Ho:YAG laser requires further optimization to prevent recurrence of small tumors.

Recent advancements in Ho:YAG laser technology have led to continuous innovations. In 2024, Yao *et al*.^17^ introduced the rotatable bi-channel *en bloc* resection of bladder tumor, which utilized a dual-channel technique to enhance precision in tumor resection. This method was successfully tested in a porcine bladder model, with no complications, such as closed-loop reflex or bladder perforation. One of the key advancements is the Moses effect, a phenomenon observed in use of Ho:YAG laser, by which laser energy delivery is optimized by momentarily separating the water layer between the laser fiber and the target tissue. This separation allows the laser pulse to reach the tissue with less initial energy loss, thereby enhancing cutting efficiency and reducing collateral thermal damage. Compared to traditional continuous laser pulses, Moses mode Ho:YAG lasers produce shorter and more focused energy bursts, leading to improved tissue ablation precision and better hemostasis.^18^,^19^ Although there are currently no reports on the use of Moses technology specifically for BCa treatment, its advantages in enhancing visualization and cutting efficiency are well-established. Future research may explore the integration of this technology into bladder tumor resection procedures to improve treatment outcomes and accelerate recovery.

The Ho:YAG laser offers significant advantages in BCa treatment, particularly in tumor resection, hemostasis, and tissue vaporization. Advancing innovative techniques based on Ho:YAG laser technology represents a promising direction toward enhancing surgical outcomes of NMIBC patients. Developments such as the Moses effect hold strong potential to enable more precise and efficient surgical procedures, thereby contributing to the continued advancement of BCa therapies.

### 2.2. Tm laser

The Tm laser, such as the Tm:YAG laser, is a laser system with an emission wavelength ranging from 1,750 to 2,220 nm. This wavelength is highly absorbed by water and penetrates only about 0.2 mm into tissue, enabling precise superficial cutting and efficient tissue vaporization. These characteristics render the Tm:YAG laser particularly suitable for urological applications, including BCa treatment.^20^ Experimental data by Wend *et al*.^20^ exhibited that the tissue ablation rate of Tm:YAG laser was significantly higher than that of KTP laser, with a rate of 6.56 g/10 min and 3.99 g/10 min, respectively. In addition, in comparison to traditional transurethral resection of the prostate, the Tm:YAG laser resulted in a 100-fold reduction in blood loss.

In 2008, the Tm:YAG laser was initially utilized for the resection of bladder malignancies, achieving favorable clinical outcomes.^21^ Its precision in cutting, in combination with minimal intraoperative complications and reduced deep thermal injury to tissues, has made the Tm:YAG laser a preferred device for endoscopic resection of bladder tumor in a relatively short period of time.^22^,^23^ An early animal study demonstrated that the tissue damage and cutting performance of Ho:YAG and Tm:YAG lasers were similar in both air and saline, with comparable hemostatic and safety profiles.^24^ Unlike Ho:YAG, the Tm:YAG laser operates in continuous mode, allowing for higher average energy output, which can lead to significant carbonization of the tissue.^25^ This increased carbonization may limit its application in certain situations.^24^ However, with its high water absorption and low penetration depth, the Tm:YAG laser has clear advantages in cutting precision and minimization of deep thermal damage, particularly when dealing with superficial tumors.^26^

Notably, the Tm:YAG laser has demonstrated significant advantages in minimizing intraoperative complications in general, and nerve reflexes in particular.^27^ In 2020, Abedi *et al*.^28^ reported that the Tm:YAG laser significantly reduced the occurrence of ONR compared to monopolar TURBT, showing superior performance. The incidence of ONR in the Tm:YAG group was 25%, substantially lower than the 63.1% observed in the monopolar TURBT group. In 2023, Diana *et al*.^27^ evaluated the effectiveness of three different power sources (bipolar resection, monopolar resection, and the Tm:YAG laser) in bladder tumor resection. The study found that the Tm:YAG group had a significantly lower occurrence of ONR (0%), in comparison to monopolar (10.2%) and bipolar (22.2%) resection groups. Furthermore, a meta-analysis comparing TURBT with Tm:YAG laser bladder tumor resection demonstrated that Tm:YAG laser significantly reduced intraoperative complications – including bladder perforation and ONR – shortened the hospital stay and irrigation time, and improved detrusor muscle recognition. In a multicenter European study^14^ involving 221 patients, Kramer *et al*. showed that the laser group experienced marginally higher post-operative hemoglobin loss compared to the traditional electrosurgery group, although the difference was not clinically significant. Furthermore, the rate of conversion back to TURBT was significantly higher in the electrosurgical group than in the laser group (26.3% vs. 1.5%).

In recent years, the Tm:YAG laser has demonstrated significant advantages in reducing recurrence rates and improving treatment outcomes in BCa. Wolters *et al*.^29^ revealed that bladder tumor resection using the Tm:YAG laser not only resulted in a lower complication rate but also achieved 100% detrusor muscle recognition. No residual transitional cell carcinoma was detected 6 weeks post-operation, providing preliminary validation of the Tm:YAG laser’s safety and efficacy in BCa treatment. Similarly, Migliari *et al*.^30^ confirmed that the Tm:YAG laser bladder tumor resection yielded a higher detrusor muscle preservation rate (100%) compared to traditional TURBT surgery. Moreover, none of the NMIBC patients treated with the Tm:YAG laser developed tumor recurrence or was positive for tumor base biopsies within a 90-day follow-up. A randomized controlled trial by Chen *et al*.^31^ demonstrated that bladder tumor resection using a 2,000-nm continuous wave Tm:YAG was superior to traditional TURBT in treating NMIBC. Although the Tm:YAG group had a higher number of T1 stage tumors (25 vs. 15 cases), which are typically associated with an increased risk of progression and recurrence, no significant difference in recurrence rates was observed between the two groups over a 18-month follow-up.

Furthermore, Tm:YAG laser bladder tumor resection failed to demonstrate an evident advantage in controlling overall recurrence rates during long-term follow-up, including recurrence within 1- and 2-year periods.^32^ However, Sun *et al*.^33^ reported that, in patients with intermediate- to high-risk NMIBC, the Tm:YAG laser significantly prolonged RFS compared to TURBT, with a lower recurrence risk (hazard ratio: 3.16; 95% confidence interval: 1.02 – 9.83). A 2024 study by Yao *et* *al*.^34^ found that, in patients with tumors larger than 3 cm, the Tm:YAG laser bladder tumor resection was significantly more effective than TURBT in terms of RFS – suggesting an improved recurrence control in larger tumors. In addition, Assem *et al*.^35^ further validated the safety and effectiveness of bladder tumor resection with Tm:YAG laser. Among 23 patients who underwent surgery, all tumors were successfully removed without major intraoperative complications, indicating that Tm:YAG laser bladder tumor resection is a safe and effective treatment alternative with a short learning curve.

The technical advantages of the Tm:YAG laser make it an ideal tool for bladder tumor resection. Interestingly, the combination of different laser technologies has been explored to further optimize surgical outcomes in BCa treatment. For instance, the combined use of the Tm:YAG and diode lasers has been shown to enhance hemostatic efficiency, reduce post-operative bleeding, and improve the accuracy of pathological analysis, thereby increasing the effectiveness of tumor resection procedures.^5^ This approach presents a promising approach to push forward Tm:YAG laser application in BCa treatment.

In summary, the Tm:YAG laser – with its excellent tissue cutting precision, low complication rates, and potential to reduce tumor recurrence – has emerged as a valuable tool for BCa resection. It offers promising opportunities to improve treatment outcomes and patient prognosis.

### 2.3. TFL

With persistent innovations in laser technology in the field of modern urology, several novel laser systems have significantly advanced BCa therapy. Among them, the TFL has emerged as a promising therapeutic tool due to its unique physical properties and clinical advantages. TFL operates in pulsed mode at a wavelength of 1,94nm, which intimately aligns with the absorption peak of water. It can also work in continuous emission modes. The absorption coefficient of water at this wavelength is approximately 114 cm^−1^ – 4 times higher than that of Ho:YAG laser – enabling more efficient absorption by intracellular water. Consequently, under the same peak power, TFL allows for more precise and rapid tissue cutting compared to other laser technologies.^36^ What is more, the theoretical penetration depth of TFL is just 0.15 mm, resulting in minimal thermal damage to surrounding healthy tissues during cutting, thereby ensuring precise and efficient cutting.^37^ Notably, compared to traditional solid-state lasers like Ho:YAG, TFL offers significant structural advantages. It utilizes a Tm-doped fiber with a diameter of 20 – 30 μm and does not require a flash-lamp-pumped, water-cooled laser crystal. This design reduces equipment complexity and heat generation while enhancing overall laser efficiency.^9^ Furthermore, during tissue resection, TFL generates fewer vapor bubbles, suggesting that it cuts tissue primarily through laser energy rather than vapor flow.

TFL also offers significant advantages in minimizing tissue carbonization.^2^ It can switch between Q-switched pulsed mode and super-pulsed mode, further enhancing its adaptability and clinical efficacy. The Q-switched pulsed mode emits high-energy laser pulses with extremely short durations (in the nanosecond range), generating high peak power while effectively controlling the thermal effect zone, thereby significantly reducing tissue carbonization. This mode is particularly suitable for surgeries that demand high precision and minimal thermal damage. In contrast, the super-pulsed mode maintains high energy output with a higher pulse repetition rate, making it efficient for medical applications asking for speed and precision – such as lithotripsy, precise tissue cutting, and minimally invasive surgeries.^9^ Compared to the Tm:YAG laser, TFL can distribute energy in a uniformly pulsed manner, maintaining a consistent relationship between peak and average power, which helps in reducing thermal damage. Studies have shown that TFL incisions are characterized by minimal carbonization, clear and intact incision edges, broad conical ablation zones, and effective coagulation. These features contribute to the superior hemostatic performance of TFL compared to Ho:YAG, which exhibited the lowest hemostasis – measured only 0.1 ± 0.2 mm – with some cases showing no coagulation.^2^,^38^

In recent years, several clinical studies have evaluated the use of TFL in BCa treatment, consistently reporting enhanced surgical safety and reduced post-operative complication rates. In a 2018 study, Rapoport *et al*.^39^ demonstrated that *en bloc* resection of bladder tumors using TFL significantly enhanced intraoperative safety – most notably by eliminating the ONR – and significantly rose detrusor muscle detection rate to 91.55%, *versus* 58.62% in the traditional TURBT group. Enikeev *et al*.^40^ conducted a prospective, non-randomized study involving 129 patients with NMIBC, and compared traditional TURBT with TFL *en bloc* resection for bladder tumor using the FiberLase U1 (NTO IRE-Polus, Russia). The TFL group demonstrated significantly higher RFS rates at both 3 months (97.2%) and 6 months (91.5%), along with an improved detrusor muscle detection rate of 91.6%. Notably, no cases of ONR or bleeding complications were observed in the TFL group, further validating the clinical benefits of TFL in bladder tumor resection. In a 2024 prospective, non-randomized controlled trial, Petov *et al*.^41^ also compared traditional TURBT with TFL bladder tumor resection using the FiberLase U1 in 129 patients with NMIBC. The TFL group achieved a significantly higher overall success rate of 93.3% compared to TURBT. In addition, specimen acquisition, including the detrusor muscle, was more efficient in the TFL group (92.8%) compared to the TURBT group (70.5%). It is worth noting that no cases of ONR were observed in the TFL group, whereas they occurred in 17.6% of the TURBT group. Although the RFS rates at 3, 6, and 12 months were comparable between the two techniques, TFL bladder tumor resection demonstrated enhanced safety, particularly for treating larger bladder tumors. Mallet^42^ further highlighted the advantages of TFL as an innovative technology in treating NMIBC. These advantages include minimal incision depth, precise cutting, enhanced hemostasis, and the absence of ballistic effects. Moreover, the average hospital stay reported lasted only 2.1 days, highlighting the potential of using TFL for outpatient surgical procedures.

Ortner *et al*.^43^ stated that the selection of laser type and settings constitutes a crucial determinant of surgical outcomes and minimizing complications in bladder tumor resection and laser vaporization. Current research highlights that Ho:YAG and TFL are among the most commonly used laser systems, with TFL increasingly replacing Ho:YAG and Tm:YAG lasers in many cases. Although there are certain differences in laser settings, these differences are generally not significant in most cases. In practice, short-pulse TFLs and long-pulse Ho:YAG lasers are typically preferred for bladder tumor resection. Continuous irrigation systems are commonly employed to minimize the risk of complications. In addition, Enikeev *et al*.^25^ reviewed various laser systems used for bladder tumor resection and concluded that water-targeted lasers – including Tm:YAG, Ho:YAG, and TFL – offer benefits compared to hemoglobin-targeted lasers such as KTP:YAG and LBO:YAG. Specifically, TFL and Tm:YAG, with their shallow penetration depths and lower peak power, have emerged as preferred modalities for bladder tumor resection, although further research is warranted to fully evaluate their benefits and limitations.

In summation, TFL has emerged as a crucial modality in BCa therapy due to its unique physical properties and significant clinical advantages. With ongoing research and continued technological advancements, TFL is expected to play an increasingly significant role in urology, contributing to improved treatment outcomes and enhanced quality of life for BCa patients.

### 2.4. GreenLight lasers

GreenLight lasers – such as KTP:YAG or LBO:YAG – are particularly effective for tissue vaporization due to their strong absorption at a wavelength of 532 nm, which targets both hemoglobin and water. Compared to other laser systems, GreenLight lasers exhibit a shallow penetration depth of approximately 0.8mm during vaporization, thereby minimizing thermal damage to surrounding healthy bladder tissues.^9^ At present, KTP and LBO lasers are widely used in soft-tissue surgeries, particularly for vaporization, demonstrating a favorable safety profile and effectiveness.^44^ A single-center randomized controlled trial further highlighted the advantages of the GreenLight laser. Although *en bloc* resection using the GreenLight laser required a longer resection time, the overall operative time did not differ significantly between groups. Notably, the GreenLight laser group showed significantly lower estimated blood loss compared to the TURBT group. In addition, resected specimens from the GreenLight laser contained a significantly higher proportion of detrusor and mucosal muscle layers than those from the TURBT group, while recurrence rates remained comparable between the two groups.^45^ These findings further confirm the GreenLight laser’s efficacy in minimizing intraoperative blood loss and enhancing resection quality.

The application of KTP GreenLight lasers in NMIBC has been gaining recognition. Tao *et al*.^46^ pioneered the use of a high-power (120W) KTP laser for treating NMIBC, demonstrating its safety and feasibility. Subsequent studies have reported consistent findings.^47^ However, compared to traditional TURBT, high-power side-firing lasers are primarily suited for tumor vaporization, which restricts complete tumor removal. This limitation often results in insufficient tissue samples for post-operative pathological assessment.^46^ To address this issue, He *et al*.^48^ developed a 30 W straight-firing KTP laser technique for transurethral bladder tumor resection. This approach enables *en bloc* tumor resection, ensuring the collection of sufficient tissues for accurate post-operative BCa diagnosis. The study reported no major intraoperative complications – including bladder rupture, hematuria, or reflexive obturator nerve contractions during surgery – and no tumor recurrence was observed during a 6-month follow-up period. Cheng *et al*.^49^ investigated the efficacy and safety of a 120 W front-firing KTP laser *en bloc* resection technique in treating NMIBC. Compared to the TURBT group, the photoselective vapoenucleation group demonstrated significantly shorter hospitalization, reduced operative time, and a lower rate of detrusor muscle absence, along with improved 1-year recurrence and tumor grade progression rates. In 2021, Tripathi *et al*.^50^ further validated the use of a 120 W side-firing KTP laser for outpatient treatment of small bladder tumors (<3 cm). Their study demonstrated several advantages of the KTP laser over bipolar TURBT, including significantly lower irrigation fluid usage (6.2 ± 0.61 L vs. 7.65 ± 0.75 L), no incidence of ONR (0 vs. 8 cases), and no perioperative complications. At the 6-month follow-up, no tumor recurrence was observed in the KTP group, whereas the TURBT group had a recurrence rate of 2.3%. The results highlight the potential of the KTP GreenLight laser in BCa treatment.

Similarly, the LBO laser has demonstrated both feasibility and safety in treating NMIBC. A prospective, non-randomized, and multicenter trial confirmed its advantages in BCa treatment. Compared to traditional TURBT, *en bloc* resection using the LBO laser was associated with a shorter surgical duration (21.46 ± 10.42 min vs. 27.48 ± 8.96 min) and a minimal reduction in hemoglobin levels (0.87 ± 0.28 g/mL vs. 1.00 ± 0.33 g/mL). Notably, no cases of ONR were reported in the LBO group, whereas nine patients in the TURBT group developed this complication. At the 36-month follow-up, no significant difference in RFS was observed between the two groups.^31^ These findings indicate that LBO laser resection not only shortens surgery time and reduces intraoperative bleeding but also lowers the risk of post-operative complications.

Zheng *et al*.^51^ evaluated the reliability and efficacy of combining high-power GreenLight laser with endoscopic mucosal resection for the treatment of primary NMIBC. All procedures were successfully completed without the need for blood transfusion, and both surgical duration and reductions in serum hemoglobin levels remained within the acceptable clinical range. However, one case of ectopic bladder was reported during the 36-month follow-up and was attributed to improper bladder irrigation. These findings highlight the promising potential of the GreenLight laser in BCa treatment and provide new insights on incorporating advanced technologies to improve treatment outcomes. In addition, the potential application of the GreenLight laser for treating type 2 MIBC has also been explored. Zhang *et al*.^52^ examined the prognostic outcomes of selective photo-vaporization using the GreenLight laser plus post-operative chemotherapy for the treatment of solitary type 2 MIBC tumors <3 cm in diameter. Their findings indicated that the laser treatment significantly reduced intraoperative blood loss and shortened post-operative hospital stays. Furthermore, the rate of short-term complications – such as bladder irritation symptoms and urinary tract infections – was comparable to that of traditional treatment modalities. No significant differences were observed between the two groups in tumor recurrence at 12, 24, and 36 months, or in RFS and overall survival outcomes.

Although the KTP laser offers several advantages, some limitations remain in its clinical application. Due to the low absorption of 532 nm wavelength by water, KTP lasers are typically delivered through side-firing technology using bare optical fibers to help manage beam direction. Both KTP and LBO lasers operate at wavelengths with minimal water absorption, and in the absence of hemoglobin, the increased attenuation length allows for deeper penetration into irrigation fluids and/or tissues – potentially posing significant risks.^25^ Compared to Ho:YAG and Tm:YAG lasers, the KTP laser tends to create a wider incision margin due to its greater tissue coagulation effect. Kramer *et al*.^13^ observed that while the KTP laser provided excellent hemostatic performance, its high cost and the relatively broad zone of thermal damage might limit its use in certain clinical contexts. Therefore, these physical properties must be carefully considered to avoid unintended damage to bladder tissues beyond the visual field.

In summary, the GreenLight laser has shown favorable safety profile and efficacy in the treatment of BCa, particularly in NMIBC and type 2 MIBC. Although there may be differences in surgical duration and resection depth, its advantages – including lower bleeding risk, fewer complications, and shorter hospital stays – highlight its potential as a promising treatment alternative. With technological advancements ongoing and clinical experience accumulating, the GreenLight laser holds strong promise for broader application in BCa treatment.

### 2.5. Diode lasers

Diode lasers have attracted mounting interest in the treatment of BCa due to their absorption characteristics at different wavelengths. Compared to other laser types, diode lasers have relatively low absorption coefficients in water. For example, at a wavelength of 980 nm, the absorption coefficient is 0.43 cm^−1^, while at 1,470 nm, it increases to 7.2 cm^−1^. These properties correspond to a theoretical tissue penetration depth ranging from 0.5 to 5.0 mm. The relatively low absorption coefficient enables deeper tissue energy transmission, rendering diode lasers particularly advantageous for treatments requiring greater penetration depth.^25^

Another significant advantage of diode lasers is their high electro-optical conversion efficiency. Compared to Ho and Tm lasers, diode lasers demonstrate superior energy utilization, which helps minimize energy loss and improve overall treatment effectiveness. In addition, their compact size and relatively low cost make them appealing for clinical applications.^6^ Moreover, diode lasers allows for improved operational control,^53^ as they do not produce the steam bubble effect – commonly associated with Ho and Tm lasers – which can cause tissue damage and reduce visibility during surgery. These characteristics highlight the promising potential of diode lasers in the treatment of bladder tumors, particularly in reducing surrounding tissue damage and improving surgical efficiency.

In the treatment of BCa, the efficacy and safety of diode lasers have been validated by several studies. Mao *et al*.^54^ evaluated the use of 980 nm diode lasers for treating primary NMIBC, reporting significant advantages over the control group in terms of bladder washout time (4.1 ± 0.6 h vs. 13.1 ± 3.1 h) and post-operative complications (occlusion reflex: 0% vs. 13.2%; delayed bleeding: 0% vs. 5.3%). However, no significant difference in recurrence rates was observed between the two groups.

Although diode lasers have been extensively used in BCa treatment, ongoing research aims to develop simpler and safer treatment approaches. Hermann *et al*.^6^ conducted the first study on photodynamic-guided 980 nm diode laser ablation for recurrent BCa under local anesthesia in an outpatient setting. The study involved 21 patients with Ta-stage, low-grade, moderate-risk bladder tumors, all of whom underwent photodynamic-guided laser ablation without sedation or analgesia. The results demonstrated that this method allowed for more accurate identification of tumors and atypical proliferative lesions that were difficult to detect under conventional white-light cystoscope, while significantly reducing treatment costs. The study provides new insights into improving patient comfort and accessibility in BCa treatment. With continued technological advancements, photodynamic-guided laser therapy may emerge as a promising option for treating low-grade bladder tumors, providing patients with a more comfortable treatment experience without the need for general anesthesia.

Further research has demonstrated that outpatient laser treatment attains excellent efficacy and patient satisfaction. In 2023, Pedersen *et al*.^7^ conducted a prospective, randomized non-inferiority trial that compared 4-month RFS rates between outpatient 980 nm diode laser coagulation for recurrent, low-grade, moderate-risk, Ta bladder tumors under local anesthesia, and traditional TURBT under general anesthesia. The diode laser group exhibited an 8% higher 4-month RFS rate compared to the TURBT group (95% CI: −8 – 24%). Patients treated with laser therapy reported lower pain scores (mean: 2.4, range: 0 – 10), while the TURBT group scored significantly higher in terms of lower urinary tract symptoms (range: 0 – 100), with an increase of 13.9 points (95% CI: 6.9 – 21.0). Moreover, the TURBT group also had an 8.1% higher incidence of minor complications compared to the laser group (95% CI: 1.0 – 14.6). Notably, 98% of patients (95% CI: 92 – 100) preferred laser treatment. These findings further support the advantages of diode lasers in the treatment of bladder tumors. In addition, for cases not requiring sedation or analgesia, outpatient diode laser ablation offers significant cost savings – approximately €140,000 per million inhabitants – compared to inpatient bladder tumor surgery.^6^

Notably, diode lasers can also be incorporated into hybrid laser systems to enhance vaporization speed, coagulation efficacy, and hemostatic performance, thereby improving both the efficiency and safety of tissue excision. Becker *et al*.^55^ compared a hybrid laser – combining a TFL with a 450 nm blue diode laser – with standalone TFL and Ho:YAG lasers for soft tissue cutting. In porcine kidney tissue models, the hybrid laser achieved the highest vaporization rate (34.4 ± 0.1 mm^3^/s) at a drag speed of 5 mm/s and produced a coagulation zone (10 ± 0.1 mm^2^) that was 2 – 3 times larger than that of the Ho:YAG laser (4 ± 0.1 mm^2^) under the same condition. In addition, hybrid lasers (*e*.*g*., combining Tm and diode lasers) demonstrated excellent performance in bladder tumor resection. Despite the use of tumor morcellation, neither hybrid nor Tm lasers impacted the pathological outcomes of tumor tissues, and the detrusor muscle remained intact. Moreover, the hybrid laser demonstrated superior hemostatic control compared to the Tm laser, supporting improved clinical outcomes for both bladder tumor excision and histopathological assessment.^5^

Diode lasers have demonstrated significant advantages in BCa treatment thanks to their low absorption coefficient, high electro-optical conversion efficiency, and compactness. No matter applied independently or integrated into hybrid laser systems, diode lasers represent a safe and effective treatment option. With technological advancements and broader clinical use, diode lasers are expected to play an increasingly important role in bladder tumor treatment, offering patients more treatment options.

## 3. Conclusion

With advancements in urological techniques, laser-based technologies have become a crucial element in BCa therapy. From Ho lasers to TFL, various systems have demonstrated significant benefits in enhancing surgical precision, safety, and patients’ quality of life. This is particularly evident with NMIBC treatment, where laser technologies enable more precise tumor resection and improved tissue preservation. Despite these advantages, several limitations linger. The high cost of laser equipment and the technical expertise required for operation continue to limit their widespread clinical adoption. In addition, while current studies have shown promising short-term outcomes, the long-term efficacy and safety of laser-based treatments remain insufficiently validated due to a lack of large-scale, randomized clinical trials. Comparative studies among different laser modalities, along with investigations into their effects on recurrence rates and overall patient survival, are critically needed. Future research should focus on integrating different laser technologies, optimizing treatment protocols, and reducing costs to improve their clinical accessibility. In addition, collaborative multicenter trials and long-term follow-up studies are essential for establishing standardized guidelines for the use of lasers in BCa treatment. Despite growing clinical adoption, large-scale studies assessing the economic costs of different laser modalities remain limited. A comprehensive cost-effectiveness analysis could provide valuable insights for optimizing treatment strategies and guiding healthcare resource allocation, particularly in NMIBC management. With ongoing technological advancements and the accumulation of clinical experience, laser-based technologies are expected to play an increasingly crucial role in the treatment of BCa.

## Data Availability

Not applicable.
